# Morphological variation of *Aphidius ervi* Haliday (Hymenoptera: Braconidae) associated with different aphid hosts

**DOI:** 10.7717/peerj.3559

**Published:** 2017-07-11

**Authors:** Cinthya M. Villegas, Vladimir Žikić, Saša S. Stanković, Sebastián A. Ortiz-Martínez, Ainara Peñalver-Cruz, Blas Lavandero

**Affiliations:** 1 Laboratorio de Interacciones Insecto-Planta, Instituto de Ciencias Biológicas, Universidad de Talca, Talca, Chile; 2 Department of Biology and Ecology, Faculty of Science and Mathematics, University of Niš, Niš, Serbia

**Keywords:** Variability, Biotypes, Geometric morphometrics, Allometry, Wing shape, Wing size, Parasitoids

## Abstract

**Background:**

Parasitoids are frequently used in biological control due to the fact that they are considered host specific and highly efficient at attacking their hosts. As they spend a significant part of their life cycle within their hosts, feeding habits and life history of their host can promote specialization via host-race formation (sequential radiation). The specialized host races from different hosts can vary morphologically, behaviorally and genetically. However, these variations are sometimes inconspicuous and require more powerful tools in order to detect variation such as geometric morphometrics analysis.

**Methods:**

We examined *Aphidius ervi*, an important introduced biological control agent in Chile associated with a great number of aphid species, which are exploiting different plant hosts and habitats. Several combinations (biotypes) of parasitoids with various aphid/host plant combinations were analyzed in order to obtain measures of forewing shape and size. To show the differences among defined biotypes, we chose 13 specific landmarks on each individual parasitoid wing. The analysis of allometric variation calculated in wing shape and size over centroid size (CS), revealed the allometric changes among biotypes collected from different hosts. To show all differences in shape of forewings, we made seven biotype pairs using an outline-based geometric morphometrics comparison.

**Results:**

The biotype *A. pis_pea* (*Acyrthosiphon pisum* on pea) was the extreme wing size in this study compared to the other analyzed biotypes. Aphid hosts have a significant influence in the morphological differentiation of the parasitoid forewing, splitting biotypes in two groups. The first group consisted of biotypes connected with *Acyrthosiphon pisum* on legumes, while the second group is composed of biotypes connected with aphids attacking cereals, with the exception of the *R. pad_wheat* (*Rhopalosiphum padi* on wheat) biotype. There was no significant effect of plant species on parasitoid wing size and shape.

**Discussion:**

Although previous studies have suggested that the genotype of parasitoids is of greater significance for the morphological variations of size and shape of wings, this study indicates that the aphid host on which *A. ervi* develops is the main factor to alter the structure of parasitoid forewings. Bigger aphid hosts implied longer and broader forewings of *A. ervi.*

## Introduction

Parasitoids are frequently used in biological control as they are considered to be highly specialized natural enemies (Godfray, 1994). By being highly specialized, released parasitoids will be the most efficient at attacking the target pest species. This reduces the possibility of environmental harm of rapidly-growing parasitoid populations migrating from crops into adjacent natural habitats (Rand, Tylianakis & Tscharntke, 2006), as has been observed for generalist predators (Duelli et al., 1990; French et al., 2001). Although several parasitoid species can exploit many hosts (Mackauer & Starý, 1967) this may not be consistent across an entire species, and different biotypes may be specialized to different hosts/environments (Stireman et al., 2006; Forbes et al., 2009). Previous studies have shown that host-associated biotypes of parasitoids from different hosts/environments can vary morphologically, behaviorally and genetically (Žikić et al., 2009; Feder & Forbes, 2010; Kos et al., 2012; Zepeda-Paulo et al., 2013). In terms of morphological features, the shape and size of their appendages have shown great promise for separating host-associated races of parasitoids. Among these, insect wings are especially relevant as they are two dimensional structures with important characteristics, in terms of adaptation and function (Wootton, 2002; Žikić et al., 2009). Previous studies have shown that the size, shape and venation of the wings can be important features to separate species and characterize populations within a single species (Sadeghi, Adriaens & Dumont, 2009). A geometric morphometrics approach is very useful for detecting minute variations in morphology of different parasitoid populations which otherwise cannot be identified easily (Villemant, Simbolotti & Kenis, 2007; Žikić et al., 2009; Kos et al., 2011). This can be of great importance because these morphological variations in wing shape could be associated with a specific environment or host-associated population of a parasitoid species.

The Chilean populations of *Aphidius ervi* (Haliday, 1834) (Hymenoptera: Braconidae) may be a good example where different host associations and environment have influenced morphology. This species is an oligophagous parasitoid associated with a number of legumes, Solanaceae and cereal aphid species. Legume feeding aphid hosts include *Acyrthosiphon pisum* (Harris, 1776), *Acyrthosiphon kondoi* (Shinji, 1938) and *Macrosiphum euphorbiae* (Thomas, 1878) with *Aulacorthum solani* (Kaltenbach, 1843) feeding on Solanaceae (Takada & Tada, 2000). Cereal aphid hosts include *Sitobion avenae* (Fabricius, 1775), *Rhopalosiphum padi* (Linnaeus, 1758), *Schizaphis graminum* (Rondani, 1852) and *Metopolophium dirhodum* (Walker, 1849) (Starý, 1993). *Aphidius ervi* was introduced in Chile in the 1970’s as part of a classical biological control strategy to minimize the damage caused by the grain aphid (*S. avenae*) on cereals and maintain the pest population at low densities in the field (Zúñiga et al., 1986). Currently, *A. ervi* is the predominant parasitoid species controlling *A. pisum* and *S. avenae*. It represents more than 94% of parasitized *A. pisum* on legumes and 38% of parasitized *S. avenae* on cereals and is considered a highly efficient biological control agent of aphids on both crops (Gerding et al., 1989; Starý et al., 1994; Zepeda-Paulo et al., 2013). The main goal of the present study is to analyze the shape and size of forewings of *A. ervi* collected in different plant/host associations, on legumes and cereals.

## Materials & Methods

### Sampled material

Aphids were collected from fields of legumes and cereals in two different geographic regions of central Chile: “Región de los Rios” (S39°51′, W73°7′) and “Región del Maule” (S35°24′, W71°40′). Parasitoids were obtained from parasitized aphids collected in the field, and after emergence carefully examined and identified. Reared samples were transferred in the growing laboratory and treated under following conditions: 20 °C, 50–60% RH, D16:N8 of photoperiod. Parasitoid wasps were put in plastic microtubes with 96% ethyl alcohol. Paraisitoid identification followed Starý (1995) for the taxonomical identification.

A total of 131 females of *Aphidius ervi* were analyzed. Parasitoids were divided into eight biotypes according to their aphid hosts and to the plant species where the aphids were found (Table 1). The biotypes used for *Acyrthosiphon pisum* were the alfalfa biotype from alfalfa (*Medicago sativa* L.), the pea biotype from pea (*Pisum sativum* L.), and the clover biotype from red clover (*Trifolium pratense* L.). Biotypes reared on cereals were the bird cherry-oat aphid (*Rhopalosiphum padi*), the rose grain aphid (*Metopolophium dirhodum*) the green-bug (*Schizaphis graminum*), and the grain aphid (*Sitobion avenae*) sampled from wheat (*Triticum aestivum* L.). Another cereal biotype is also the grain aphid (*Sitobion avenae*) which was collected from oat (*Avena sativa* L.) (Table 1).

**Table 1:** *Aphidius ervi* material sampled and biotype definitions.

Aphid host	Host-plant	No of specimens	Biotype
*Acyrthosiphon pisum*	alfalfa	29	*A. pis_alfalfa*
*Acyrthosiphon pisum*	pea	28	*A. pis_pea*
*Acyrthosiphon pisum*	red clover	14	*A. pis_clover*
*Metopolophium dirhodum*	wheat	10	*M. dir_wheat*
*Rhopalosiphum padi*	wheat	10	*R. pad_wheat*
*Schizaphis graminum*	wheat	13	*Sc. gra_wheat*
*Sitobion avenae*	oat	14	*S. ave_oat*
*Sitobion avenae*	wheat	13	*S. ave_wheat*
**Total**		**131**	

### Geometric morphometrics

To conduct the geometric morphometrics analysis, we applied two-dimensional landmark-based methods (Bookstein, 1986; Bookstein, 1991). Right forewings of each female parasitoid were removed and mounted in Neo Mount (Merck) following the procedure described in Žikić et al. (2009). Forewings were recorded using an OPTIKA SZN (45x) stereoscopic compound microscope with a mounted 5-megapixel photographic camera using software Optika Vision Pro v2.7. Using the geometric morphometrics method (Zelditch et al., 2004) we determined and quantified morphological variations of wing size and shape in different *Aphidius ervi* biotypes.

**Figure 1:**
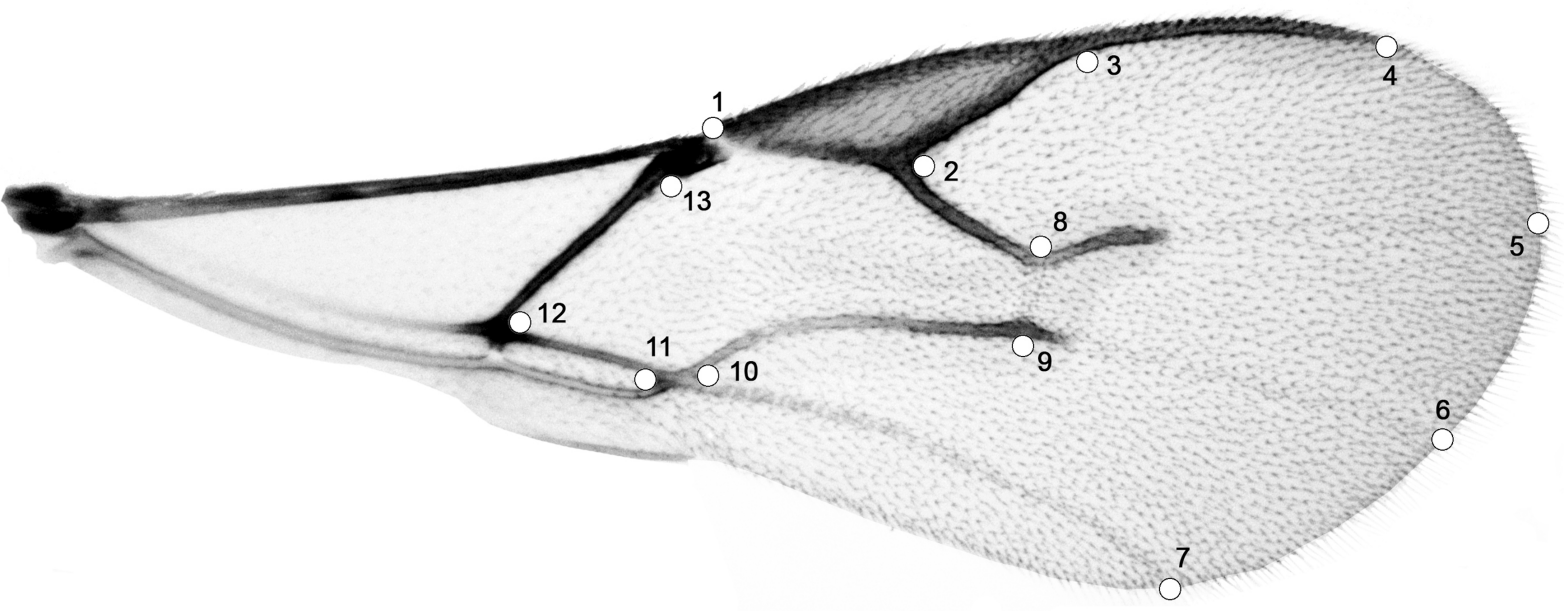
Right forewing of *Aphidius ervi*; set of 13 specific landmarks.

**Table 2:** Description of specific forewing landmarks used in the analyses. Wing vein terminology follows Wharton, Sharkey & Michael (1997).

Landmark number	Landmark definition
1	Beginning of stigma
2	Corner at the middle of stigma and r vein
3	End of stigma
4	End of metacarpus
5	Projection of RS vein on the edge of wing
6	Projection of M vein on the edge of wing
7	Projection of CU vein on the edge of wing
8	Corner of RS and r-m veins
9	Corner of M and r-m veins
10	Corner of m-cu and 1CU veins
11	Corner of 1CU and 1A veins
12	Corner of 1M and 1CU
13	Beginning of parastigma

Eight different aphid-host/plant-host associations were used for morphological characterization of *A. ervi* biotypes (Table 1). To analyze the variation in wing shape of parasitoids, 13 specific landmarks were scored for each forewing. Positioned landmarks were digitized using software TpsDig v2.16 (Rohlf, 2010) (Fig. 1 and Table 2). Using a generalized procrustes analysis (an analysis that allows morphotype separation) all variations due to scale, orientation and position of the 13 landmark configurations were eliminated (Rohlf & Slice, 1990; Bookstein, 1991). Centroid size (CS) was calculated for each forewing, indicating the dispersion of the landmarks from the centroid; this parameter is used as a relative indicator of the wing size. Size variation among forewings (obtained on the basis of the CS) was examined using the analysis of variance (ANOVA) performed on the centroid size. To see if there were some correlations between the wing size and shape, we performed a regression test between the CS and procrustes coordinates (PC) scores (Žikić et al., 2010). Discriminant analysis using the residuals of the regression test was performed to determine if any of the procrustes distances were statistically significant. The latter to understand if changes in wing shape were caused by changes of the wing size. Resultant shape variables were also analyzed using multivariate analysis of variance (MANOVA) performed on eigenvalues of the PC scores. The MorphoJ software was used to analyze and visualize shape changes described by canonical axes (Klingenberg, 2011). Principal component analysis (PCA) was used to analyze variability in wing shape among the specimens investigated. This analysis allowed us to group the different biotypes studied. The differences in wing shape were visualized using canonical variate analysis (CVA) in order to observe the variability among the *A. ervi* biotypes (Rohlf, 2010) (Fig. S2). The centroid sizes were obtained using the MorphoJ v1.06b software (Klingenberg, 2011). For the visualization of wing shape changes between the analysed biotypes, outline drawings consisting of a series of lines that are in a specific relation to the arrangement of the landmarks were created. MorphoJ uses the thin-plate spline method to produce a deformation of the drawing so that the arrangement of landmark points matches the configurations that are to be visualized (see Klingenberg, 2011). All statistical tests concerning analysis of variance (ANOVA) and multivariate analysis of variance (MANOVA) were performed in Statistica 7.0 (StatSoft, Tulsa, OK, USA).

## Results

**Figure 2:**
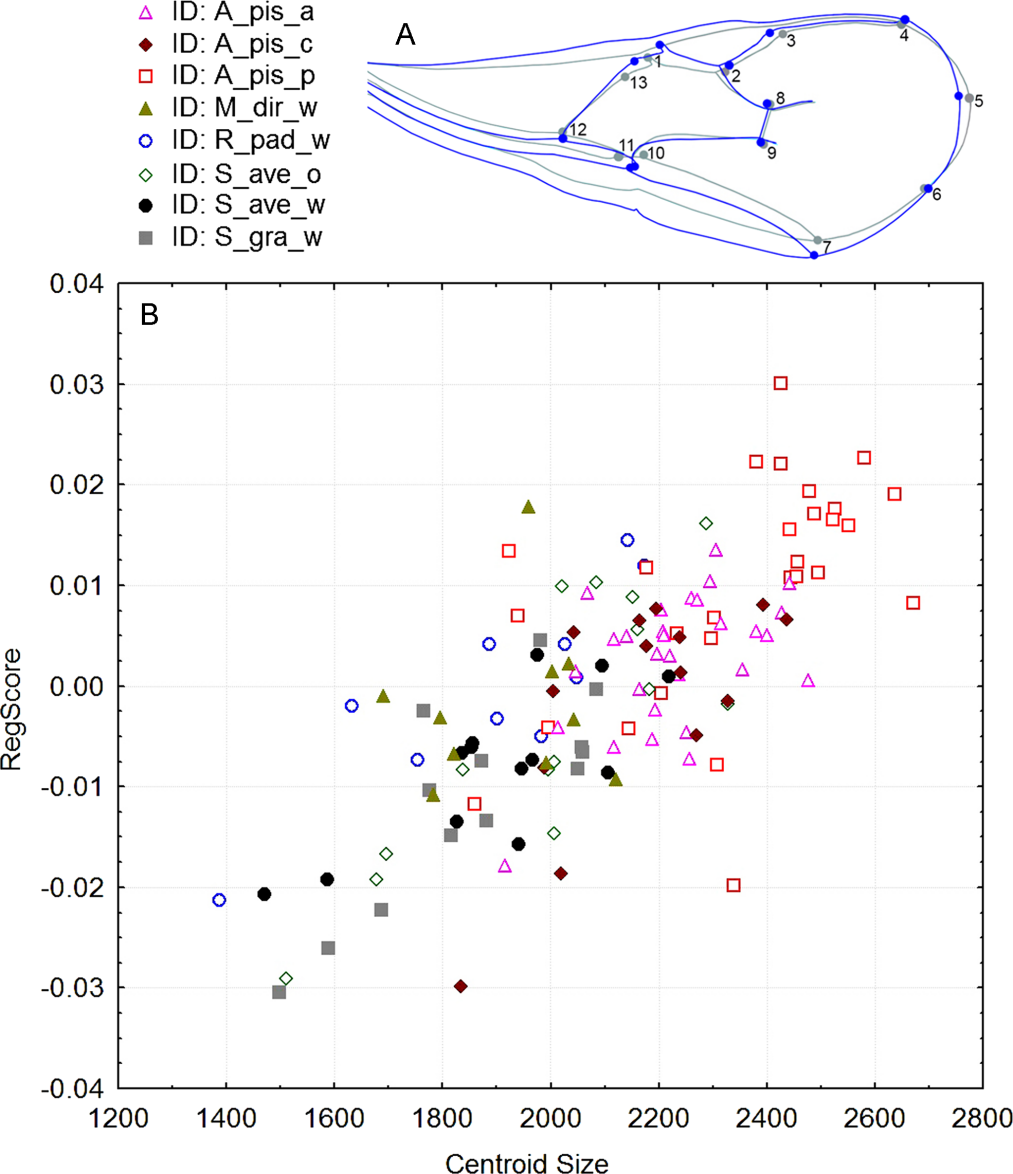
(A): Figure of forewing shape changes in A_pis_p biotype. The blue line represents the largest wing shape analyzed, while the gray line represents the average wing shape. (B): The regression results of the centroid size (CS) and PC scores (permutation test against the null hypothesis of independence, *P*-value: <0.0001). The included biotypes were *Acyrthosiphon pisum* from alfalfa (A_pis_a), *A. pisum* from red clover (A_pis_c), *A. pisum* from pea (A_pis_p), *Metopolophium dirhodum* from wheat (M_dir_w), *Rhopalosiphum padi* from wheat (R_pad_w), *Sitobion avenae* from oat (S_ave_o) and wheat (S_ave_w) and *Schizaphis graminum* from wheat (S_gra_w).

Significant differences in shape were observed with the procustes ANOVA analyses (*F* = 17.30; *df* = 7; *P* < 0.000001). However, according to the PCA, the variability explained by the first three axes was rather low; all three explain 50.6% of the total variability (Fig. S1). Forewing size, and shape were significantly different using the PC scores (MANOVA: Wilks’ λ = 0.112737; *F* = 1.74; *df* = 154; *P* < 0.000001). Considering that all statistical tests of variance were statistically significant, we performed a canonical variate analysis (CVA) to observe the variability among the *A. ervi* biotypes (Fig. S2). However, there was no conspicuous grouping of the biotypes into discrete morphotypes. The first canonical axis (CV1) explains 38.4%, while the second axis (CV2) explains only 23% of the total variability. To see if there was some correlation between the wing size and shape we performed the regression test between the centroid size and PC scores. The statistical test showed that the wing shape is clearly correlated with the wing size (*P*-value: <0.0001; Fig. 2). The percentage of the wing shape variability explained by this regression test is only 6.78% (% predicted: 6.7783%), therefore the wing size has a small contribution to variations in wing shape. The largest wings were of the specimens from the biotype *A. pis_pea*, while the smallest were those from *A. ervi* parasitizing *S. avenae* on wheat (biotype *S. ave_wheat*) and on *S. graminum* also on wheat (Fig. 2).

**Figure 3:**
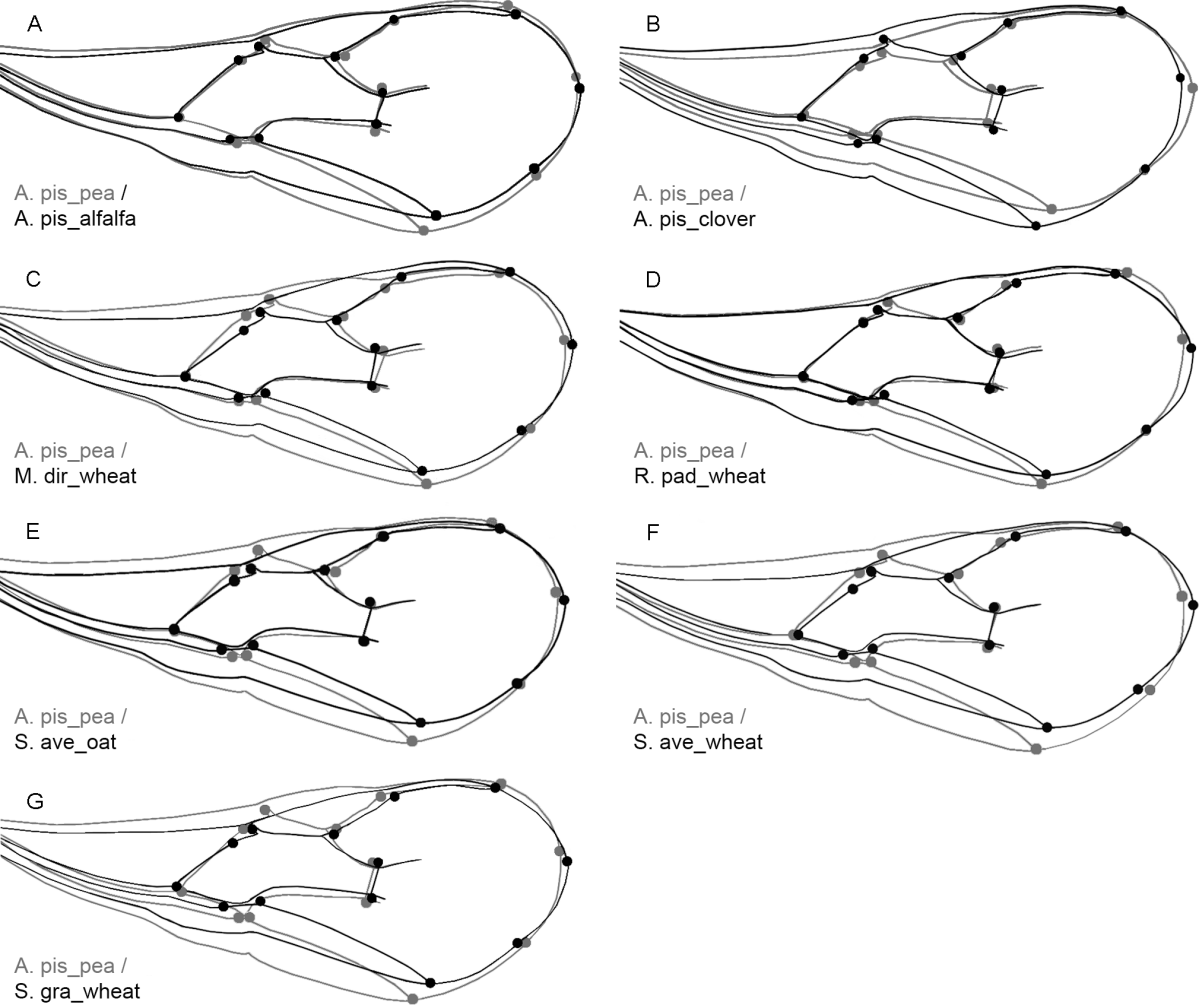
Outline-based comparison of the wing shape between the biotype A. pis_pea and the rest of the seven biotypes: *Acyrthosiphon pisum* wing shape from pea (A. pis_pea) was compared to *A. pisum* from alfalfa (A. pis_alfalfa) (A), to *A. pisum* from red clover (A. pis_clover) (B), to *Metopolophium dirhodum* from wheat (M. dir_wheat) (C), to *Rhopalosiphum padi* from wheat (R. pad_wheat) (D), to *Sitobion avenae* from oat (S. ave_oat) (E), to *Sitobion avenae* from wheat (S. ave_wheat) (F) and to *Schizaphis graminum* from wheat (S. gra_wheat) (G). Shape differences are the results of discriminant analysis (DA). The scale factor is increased by 5. The grey outline represents the biotype *A. pis_pea*; the black outline represents the other biotypes compared.

Considering that the regression result was statistically significant (*P*-value: <0.0001) we performed a discriminant analysis (DA) using the residuals to clarify the influence of the wing size on its shape. This particular analysis showed that none of the procrustes distances were statistically significant (*P*-value: >0.05), suggesting that although small there are some morphological changes caused by the variation in size. Given that the biotype *A. pis_pea* has the largest wings, we wanted to visualize how the wings of all other *A. ervi* biotypes change in relation to this particular biotype (*A. pis_pea*) using an outline-based geometric morphometric method (Fig. 3). The changes between the biotype *A. pis_pea* and the other six can be seen in Fig. 3.

The least observed changes of the wing shape were detected between the following pairs: *A. pis_pea*/*A. pis_alfalfa*, *A. pis_pea*/*A. pis_clover* and *A. pis_pea*/*R. pad_wheat* (see relations in Fig. 3 and Fig. S2). More conspicuous changes were visible for the comparison between *A. pis_pea*/*S. ave_oat*, and *A. pis_pea*/*S. ave_wheat*. The latter changes are due to the narrowing of the wing in the two biotypes (*S. ave_oat* and *S. ave_wheat*). The greatest difference observed was between the biotype *A. pis_pea* and *S. gra_wheat*: this biotype has the narrowest wing in relation to *A. pis_pea* (Fig. 3 and Fig. S2).

## Discussion

*Aphidius ervi* is known to attack economically important pests worldwide and in the Chilean agricultural landscapes it is considered a successful example of classical biological control of legume and cereal aphids (Starý, 1993; Starý et al., 1993; Rojas, 2005). Although it is very efficient in parasitizing target aphid pests, it has not been observed attacking native aphid species in shared environments (e.g: *Uroleucon* species developing on native plants in and around agricultural valleys in Chile) (Zúñiga et al., 1986; Starý, 1993). Many studies have shown heritable host fidelity and have hypothesized the possibility of different host associated biotypes. However, recent studies of Bilodeau et al. (2013) and Zepeda-Paulo et al. (2013) using population genetics suggest that in both North America and Chile there are no specialized races or biotypes on different aphid-host species, revealing high gene flow between these parasitoid populations.

In a recent study, it has been shown that the parasitoid genotype can have a stronger influence on wing shape than developing on a different parasitoid host species (Parreño et al., 2016). These authors used five asexual lines of *Lysiphlebus fabarum* (Marshall, 1896) (Braconidae) and four aphid hosts, and they found by using the procrustes coordinates on wings that the lineages acted as a better grouping factor compared to the parasitoid aphid-host variable. In this study, we did not discover any distinctive morphological features that could differentiate the Chilean populations of *A. ervi*. However, the significant narrowing of the wings observed for the *S. ave_wheat* and *S. gra_wheat* biotypes when compared to the *A. pis_pea* biotype is an indication of environmental and ecological effects particular to each parasitoid population (Fig. 3). The low genetic variability observed between specimens of *A. ervi* from different aphid host and locations suggests a high gene flow between parasitoid populations, with the result of no local adaptation or host associated races (Zepeda-Paulo et al., 2016).

Comparing the allometric relationships of wings among tested biotypes, it was found that the smallest wings were from *S. gra_wheat*, while the biggest wings were from *A. pis_pea* biotype (Fig. 2). This particular variability in wing size has morphological effects on the wing shape, causing the subtle changes among analyzed biotypes (Fig. 3). Therefore, this particular wing from the *A. pis_pea* biotype was used to compare it with the wings of the other seven biotypes (Fig. 3).

Conspicuous differences of the wing size and shape between *A. pis_pea* and other biotypes were clearer for those biotypes reared on cereals, compared to those biotypes from legumes. The specimens of this particular biotype have generally larger forewings than the other biotypes and are broader in the middle and the distal part (Figs. 2 and 3). The least deviation from the average wing constructed is observed for the *R. pad_wheat* biotype, where the differences were less noticeable (Fig. 3). This could be the effect of the aphid host size, because *Acyrthosiphon pisum* is rather a large aphid in comparison to *Rhopalosiphum padi.* Certainly, the biotypes reared from *Acyrthosiphon pisum* (*A. pis_alfalfa*, *A. pis_clover* and *A. pis_pea*) have the largest wings independent of the aphid clone (host-plant). Compared to all other analyzed aphid species (≤3 mm), which are hosts of *A. ervi*, *A. pisum* is the biggest (≤5.5 mm) (Blackman & Eastop, 2008).

Parasitoids with smaller wings emerged from aphid hosts feeding on cereals (wheat and oats), while from *A. pisum* feeding on legumes (alfalfa, clover and pea) the emerged individuals had larger wings. Although the effects of plant species on the *A. ervi* biotypes were not addressed here, this should not be completely neglected. Some evidences suggest that the preference of *A. ervi* biotypes toward plant/aphid host volatiles will eventually lead them to the adequate aphid host (Daza-Bustamante et al., 2002). Host and plant preferences could cause physiological changes in *A. ervi* as suggested by Cameron, Powell & Loxdale (1984). This could explain the variability in body size of parasitoids and the morphological differentiation of the forewings among the analyzed biotypes. The influence of host/plant association on morphological differentiation of forewings has been also shown in other studies of braconid wasps; e.g., biotypes from the genus *Eubazus* (Nees, 1814), a parasitoid of the conifer bark weevil (Villemant, Simbolotti & Kenis, 2007) or *Lysiphlebus fabarum* (Parreño et al., 2016).

Variations of the shape of insect wings are known to affect flight ability, which in turn could alter the host and mate allocation (Kölliker-Ott, Blows & Hoffmann, 2003). Betts & Wootton (1988) studied the effects of wing structure on the flight of six butterfly species and showed that there was a correlation between flight performance and wing shape. Additionally, studies have described how the wing shape can alter predation success by dragonflies (Combes, Crall & Mukherjee, 2010) and the ability of damselflies to avoid predation by passerine birds (Outomuro & Johansson, 2015). More specifically, parasitoids are affected by changes in wing size and shape. The wing size and shape of *Trichogramma brassicae* (Bezdenko, 1968) and *Trichogramma pretiosum* (Riley, 1879) as egg parasitoids, increase the ability to locate host eggs. Differences in wing size and shape were found between parasitoids obtained from field conditions compared to those parasitoids that were reared in the laboratory (Kölliker-Ott, Blows & Hoffmann, 2003). Authors suggest that wing shape and wing size can be reliable predictors of field fitness for these parasitoid species. In the present study, the biotypes of *A. ervi* emerged from *A. pisum* had larger and broader forewings compared to the other studied biotypes. These differences of wing shape and size could affect the fitness of *A. ervi* and its ability to find aphid hosts. Further research to determine the most suitable aphid host for *A. ervi* to increase its fitness will lead to enhanced rearing conditions for *A. ervi* and consequently, will improve any future inundative biological control strategies with this parasitoid.

## Conclusion

Given the low genetic variability of *Aphidius ervi* in Chile, the main factor affecting morphological variations of *A. ervi* forewings is their aphid host. Forewing shape variability is partly influenced by allometric effects. The greatest difference in *A. ervi* wings among aphid hosts were observed between *A. pisum* and the cereal aphids in general.

##  Supplemental Information

10.7717/peerj.3559/supp-1 Figure S1 Principal component analysis Distribution of *Aphidius ervi* biotypes in the morphospace defined by PC1 and PC2 axes. The total variability explained for PC1 + PC2 = 37.39%.

10.7717/peerj.3559/supp-2 Figure S2 Cannonical variate analysis Distribution of *Aphidius ervi* biotypes in the morphospace defined by CV1 and CV2 axes. The total variability explained for CV 1 + CV 2 = 61.4%.

## Additional Information and Declarations

### Data Availability

The following information was supplied regarding data availability: Figshare: https://figshare.com/articles/Ervi_morphoj/4650271.
