# Supplementary figures and images for: Morphological variation of Aphidius ervi Haliday (Hymenoptera: Braconidae) associated with different aphid hosts
Source: PeerJ. 2017 Jul 11;5:e3559. doi: 10.7717/peerj.3559 (PMC5508808; doi:10.7717/peerj.3559)

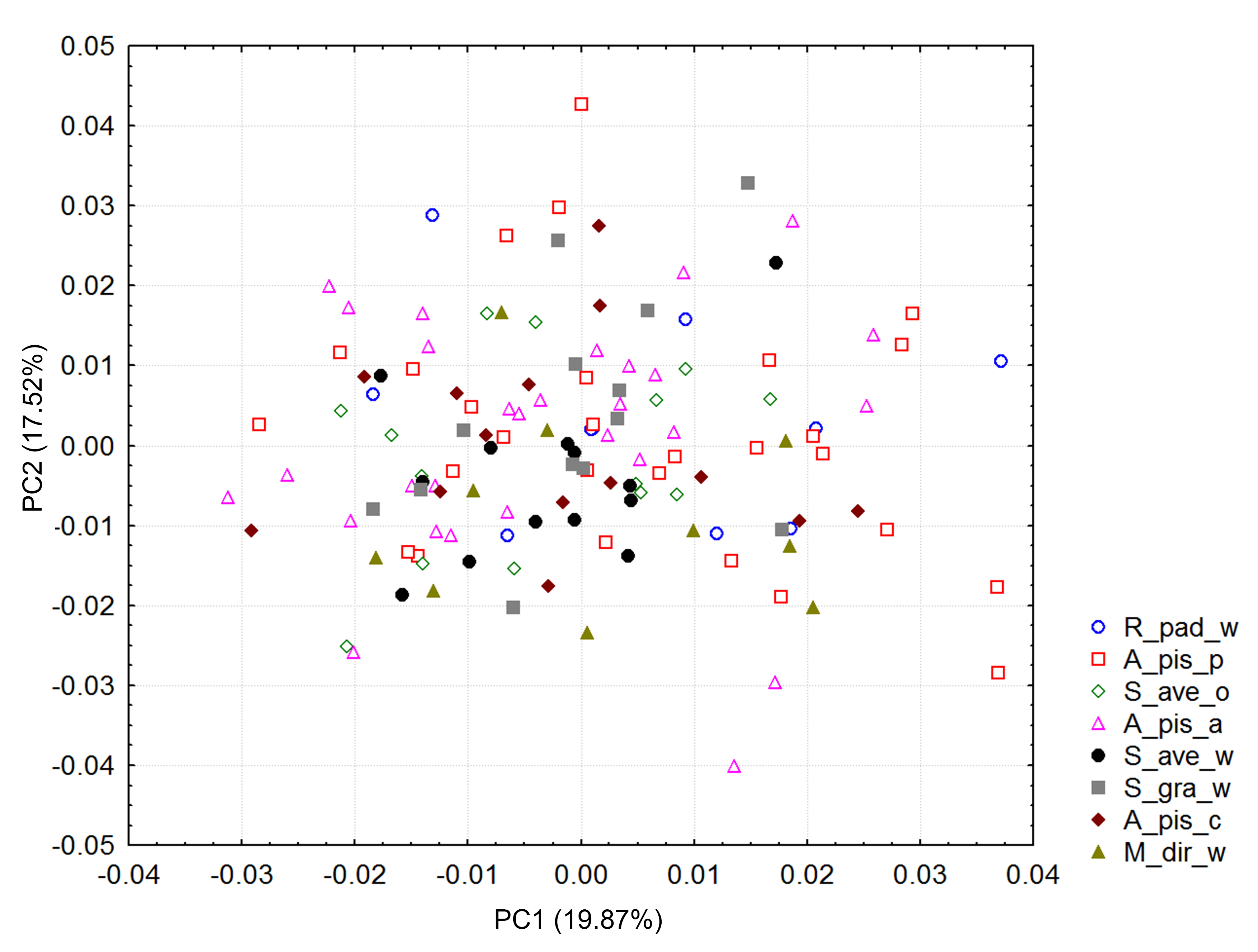

Supplement: Figure S1 — Distribution of Aphidius ervi biotypes in the morphospace defined by PC1 and PC2 axes. The total variability explained for PC1 + PC2 = 37.39%. [file peerj-05-3559-s001.png]

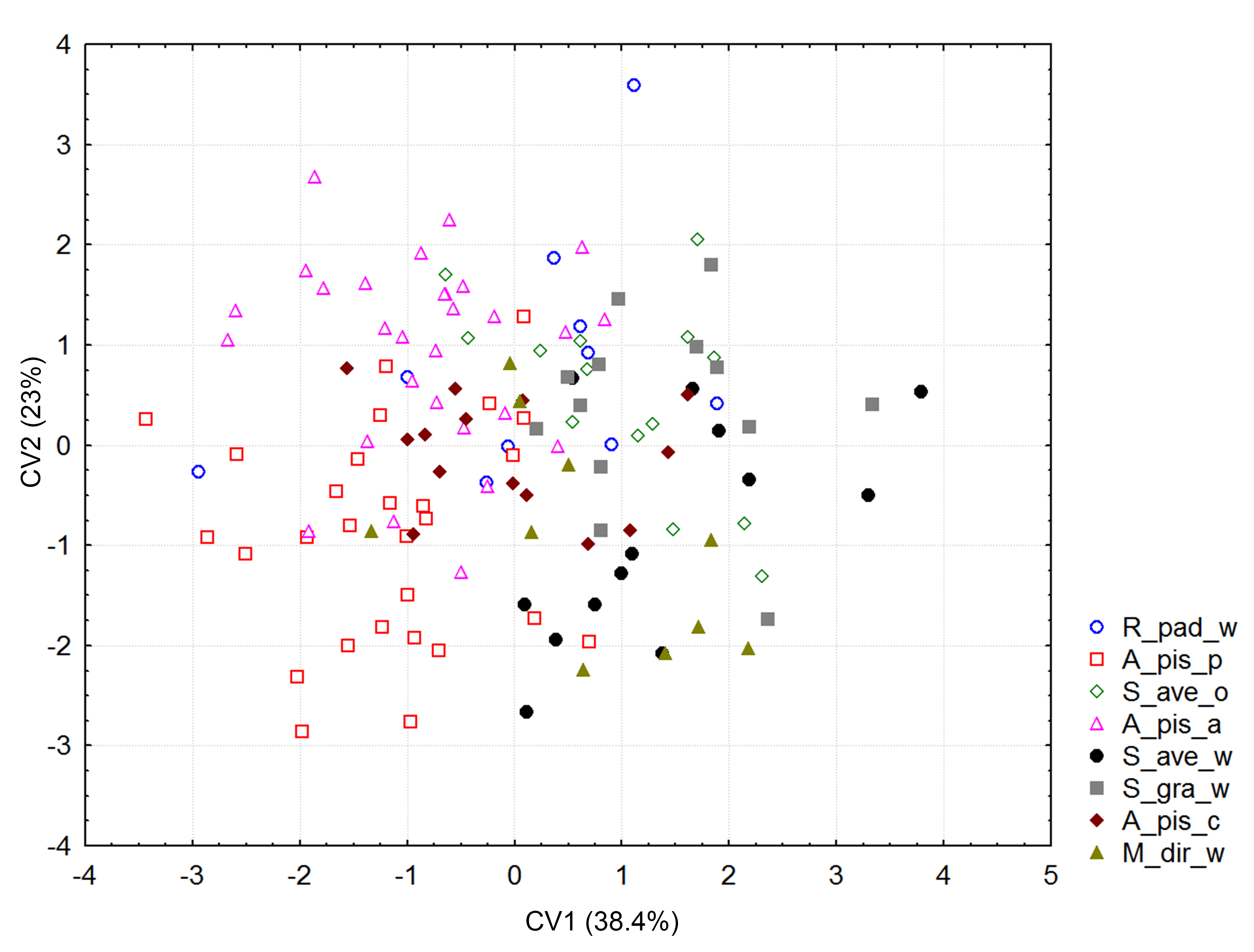

Supplement: Figure S2 — Distribution of Aphidius ervi biotypes in the morphospace defined by CV1 and CV2 axes. The total variability explained for CV 1 + CV 2 = 61.4%. [file peerj-05-3559-s002.png]
